# Hospital-Acquired Acute Kidney Injury in Noncritical Care Setting: Clinical Characteristics and Outcomes

**DOI:** 10.1155/2022/7077587

**Published:** 2022-05-16

**Authors:** Maggie Tso, Kamal Sud, Connie Van, Abhijit Patekar, Wubshet Tesfaye, Ronald L. Castelino

**Affiliations:** ^1^The University of Sydney, Faculty of Medicine and Health, School of Pharmacy, Camperdown, Australia; ^2^The University of Sydney Nepean Clinical School, Faculty of Medicine and Health, Kingswood, Australia; ^3^Renal Medicine, Nepean Hospital, Kingswood, Australia; ^4^Transplantation Medical Unit, Westmead Hospital, Westmead, Australia; ^5^The University of Canberra, Health Research Institute, Faculty of Health, Canberra, Australia; ^6^Department of Pharmacy, Blacktown Hospital, Blacktown, Australia

## Abstract

**Background:**

There is limited Australian data on the incidence and outcomes of hospital-acquired acute kidney injury (HA-AKI) in noncritically ill patients.

**Aims:**

This study aimed to characterise HA-AKI and assess the impact of nephrology consultations on outcomes.

**Methods:**

A retrospective cohort of all noncritically ill patients with HA-AKI admitted to a large tertiary hospital in 2018 were followed up from hospital admission to discharge. HA-AKI was defined using the Kidney Disease Improving Global Outcomes (KDIGO) criteria. The primary outcome of this study was the clinical characteristics of patients who developed HA-AKI and the difference in these characteristics by nephrology consultation.

**Results:**

A total of 222 noncritically ill patients were included in the study. The mean age of included patients was 74.8 ± 15.8 years and 57.2% were females. While most patients (92%)were characterised to have KDIGO stage 1, 14% received a nephrology consultation, and 80% had complete or partial recovery of kidney function at discharge. Lower recovery rates (65% versus 83%, *P* = 0.022), longer hospitalisations (10 versus 5 days, *P* = 0.001), and higher serum creatinine values on discharge (152 versus 101 *μ*mol/L, *P* < 0.001) were associated with receipt of nephrology consultation. There was no difference in mortality rates (13% versus 11%, *P* = 0.754) between those with and without nephrology consultation.

**Conclusions:**

Our findings indicate that signficant proportion of noncritically ill patients experience mild form of AKI and have good recovery of kidney function during hospitalisation. Although severity of AKI and length of hospitalisation were associated with nephrology interventions, large scale study is required to understand the impact of such interventions on clinical outcomes, such as hospital readmission and mortality.

## 1. Background

Acute kidney injury (AKI) is a common clinical syndrome in hospitalised patients and is increasing in incidence globally [1, 2]. It is often caused by reduced renal perfusion resulting from hypotension, hypovolemia, medications, recent surgery, radiographic contrast media, or sepsis [2, 3]. AKI is diagnosed clinically based on a rise in serum creatinine (SCr) and/or a decline in urine output, caused by an abrupt reduction in the glomerular filtration rate (GFR).

Hospital-acquired acute kidney injury (HA-AKI) has been independently associated with increased length of hospital stay, health care costs, risk of developing chronic kidney disease (CKD), early and long-term mortality, and need for ongoing posthospitalisation care [2, 4–6]. HA-AKI is the eighth most common hospital-acquired complication in Australia, with each admission involving HA-AKI presumed to incur approximately $56,000 in extra costs [1, 2]. A national snapshot between 2012 and 2013 captured that 1.6% of hospitalisations were due to AKI as the principal and/or an additional diagnosis [1]. In 2015–16, approximately 980 people in Australian public hospitals, which is equivalent to 2.2 per 10,000 hospitalisations, developed severe AKI that required haemodialysis [2].

Multiple studies have established the necessity of reducing the risk of developing HA-AKI and improving outcomes and prognosis [7–10]. Although the majority of inpatient AKI occurs in the general hospitalised population [11], most studies on AKI are conducted in the intensive care unit (ICU) context. Australian hospital ICUs tend to be of the closed ICU model, where the intensive care specialists, who have had training and experience in managing this condition, take responsibility for all decision making for the patients in the intensive care setting [12]. This difference in the nature and level of care received by patients in the ICU as opposed to those outside underpins the need to understand HA-AKI in the non-ICU setting, with a view to identify scope for future interventions. This is important because early detection of AKI events may lead to improved clinical outcomes in hospitalised patients [7, 13]. Also, the limited data in the Australian context in relation to HA-AKI emphasises the need to understand the characteristics of HA-AKI and the impact of nephrology involvement in improving outcomes in this patient group. We hypothesised that HA-AKI in non-ICU settings would be mild, largely preventable, and reversible as well as associated with shorter hospitalisation and lower incidence of in-hospital morality, especially upon early intervention by nephrologists.

Hence, the main objectives of this study are to determine the clinical characteristics of HA-AKI and assess its severity in noncritically ill adult patients. The study further aims to evaluate the impact of nephrology consultations on recovery from AKI, length of hospitalisation, and in-hospital mortality.

## 2. Methods

### 2.1. Study Design and Participants

This retrospective audit included data pertaining to patients admitted to a large tertiary care university teaching hospital in New South Wales, Australia, between 1^st^ January and 31^st^ December 2018. Adult patients (≥18 years) admitted to a hospital for over 24 hours with at least two SCr measurements within 7 days of one another were included in the study. Patients were excluded if they had kidney failure, were on any kidney replacement therapy or managed conservatively (inclusive of palliative care), were diagnosed with AKI on admission, had first SCr on admission of >300 *μ*mol/L, or were suspected of having community-acquired AKI as per the Duff and Murray's conceptual model of the proposed retrospective staging of AKI on admission [14]. Patients were also excluded when during initial screening, the differences in SCr values were clearly from two different hospital admissions, the patients were under 18 years of age, they were identified to have falsely elevated SCr where there appears to be an increase in SCr while the estimated GFR remains >90 mL/min/1.73 m^2^, and where 50% increase from the first SCr measure on admission exceeds the KDIGO AKI definition of within 7 days. This study was approved by the Institutional Human Ethics Committee.

### 2.2. Data Collection

Demographic characteristics, comorbidities, and relevant clinical information including major risk factors for AKI [15] were extracted from the electronic medical records. Relevant coexisting medical conditions were categorised based on the International Classification of Diseases, 10^th^ Edition (ICD-10) [16]. Medications were categorised as per the Anatomical Therapeutic Chemical (ATC) classification system [17]. AKI was staged for severity according to the Kidney Disease Improving Global Outcomes (KDIGO) criteria based on the SCr (Table 1) [18]. The severity of AKI was recorded along with the extent of recovery of kidney function at the time of hospital discharge and including any in-hospital mortality. Information on reason for hospital admission, length of hospitalisation, time from admission to AKI occurrence, and duration from AKI development to discharge were recorded. The treating team at the time of AKI development (categorised to medical or surgical) and surgical procedures performed during hospitalisation were recorded. Time to nephrology consultations, if any, during hospitalisation was also recorded. Documentation of the primary aetiology of AKI, including whether it was a single aetiology or multifactorial, and the need for dialysis either during hospitalisation or continuing at discharge were all recorded.

### 2.3. Definitions

The KDIGO 2012 definition of AKI was applied to all hospital admissions that occurred during the study period, and patients were selected if there was an increase in SCr of ≥26.5 *µ*mol/L within 48 hours or a 50% increase from presumed baseline known or presumed to have occurred within the prior 7 days [18]. The first/admission SCr was used as the baseline reference value and was confirmed to be representative of true baseline by review of results in the preceding 12 months. For patients with unknown baseline values, an episode of AKI could be inferred because of the subsequent clinical course of SCr measurements, which further enabled approximation of baseline SCr and confirmation of true AKI. The KDIGO staging of AKI criteria was used as a basis for defining recovery. “Complete” recovery from AKI was defined as the absence of AKI criteria; “partial” recovery was defined as a fall in AKI stage or reduction of SCr by <26.5 *μ*mol/L [18–20]. If the criteria for “complete” or “partial” recovery were not met, the case was defined as “no” recovery. SCr at hospital discharge was used to calculate and report recovery.

### 2.4. Outcomes

The primary outcome of this study was the clinical characteristics of patients who developed HA-AKI and the difference in these characteristics by nephrology consultation. Additional outcomes included differences in recovery rates from AKI, length of hospital stay, and mortality rates based on referral to nephrology consultation.

### 2.5. Statistical Analysis

Statistical analysis was performed using IBM SPSS Statistics for Windows, Version 25.0 (Armonk, NY: IBM Corp.). Descriptive statistics were reported using mean and standard deviation (SD) or median and interquartile range (IQR) for continuous variables depending on the normality of data distribution, while frequencies and proportions were reported for categorical data. Outcomes for patients with versus without nephrology consultation were compared using Chi-square tests or Fisher-Exact test for categorical variables, and independent *t*-tests and Mann–Whitney *U* tests for continuous variables. For all analysis, statistical significance was set at *P* value of <0.05.

## 3. Results

**Figure 1:**
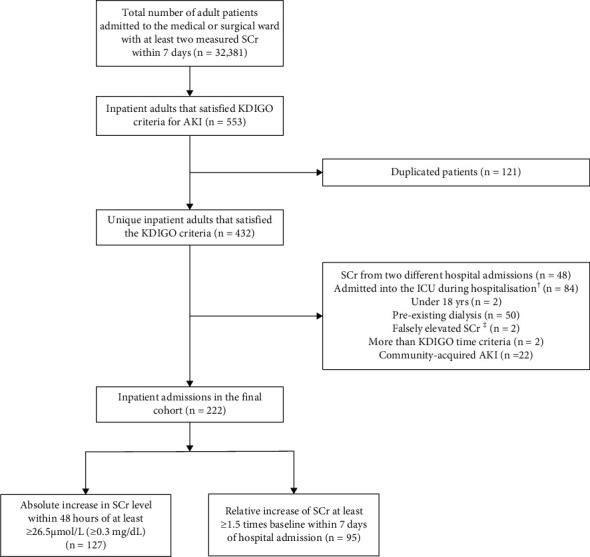
Flow diagram for the study cohort. ^†^Intensive care specialists take responsibility for all decision making for the patients in the ICU setting and specific impact of nephrology consultation being inconclusive; ^‡^where there has been an increase in SCr while estimated GFR remains >90 mL/min/1.73 m^2^.

A total of 32,381 adult patients were admitted during the study period with at least two SCr measurements within 7 days of one another. A total of 553 patients were considered for evaluation based on the inclusion and exclusion criteria. Two hundred twenty-two patients met the KDIGO's AKI criteria and were eligible for this study (Figure 1). The baseline demographic and clinical characteristics of the cohort are presented in Table 2. The mean (SD) age was 74.8 (SD = 15.8) years, and 57.2% (*n* = 127) of patients were female. The most common reasons for presentation at the hospital were confusion (*n* = 60; 19.7%), urinary incontinence (*n* = 25; 8.2%), falls (*n* = 22; 7.2%), syncope (*n* = 15; 4.9%), and shortness of breath (*n* = 13; 4.3%). The median (IQR) number of diagnosed medical conditions was 7 (4–10), with diseases of the circulatory system (*n* = 426; 26.8%), endocrine, nutritional, and metabolic diseases (*n* = 254; 16%), and diseases of the digestive system (*n* = 154; 9.7%) identified as the most common medical conditions. Medications acting on the cardiovascular system were the most prescribed (*n* = 505; 32.4%), followed by those acting on the alimentary tract and metabolism (*n* = 440; 28.3%) and medications for the nervous system (*n* = 189; 12.1%).

Most patients were admitted under the medical specialty ward (*n* = 194; 87.4%). Hypertension (73.9%), advanced age (56.8%), and the use of nephrotoxic medications (54.1%) were the three topmost risk factors for AKI in the included patients. There was a lack of documentation on whether the AKI had a single aetiology or multiple aetiologies or its primary cause in more than half of the included patients (61.3%). Of those with documented causes of AKI, a single aetiology was reported in 50 (22.5%) patients, with pre-renal AKI identified as the most common primary aetiology (*n* = 50; 22.5%).

A total of 31 (14%) patients were either admitted under renal medicine or had a nephrology consultation during the study period. Of these, 23 patients were referred for nephrology consultation; 15 (65.2%) were consulted in less than 48 hours of AKI detection. No significant differences were observed in terms of baseline characteristics among patients who received a nephrology consultation and those who did not (Table 2).

More than half of the patients (*n* = 127; 57.2%) had an absolute increase in SCr of at least ≥26.5 *μ*mol/L (≥0.3 mg/dL) within 48 hours of hospital admission. While the average (SD) baseline SCr of the study population was 104 *µ*mol/L (SD = 50.3), this increased to an average of 156 *μ*mol/L (SD = 69.2) with a maximum SCr of 172.6 *μ*mol/L (SD = 103.5) during the AKI episode. Baseline SCr and SCr at detection of AKI were significantly higher in patients who received a nephrology consultation than those who did not (median (IQR) of 127 (89–175) *μ*mol/L versus 86 (68–121) *μ*mol/L (*P* < 0.001), respectively) and (214 (157–281) *μ*mol/L versus 135 (105–172) *μ*mol/L) (*P* < 0.001), respectively). In addition, the peak median SCr value was also significantly higher in patients who received nephrology consultation, (268 (192–434) *μ*mol/L), as compared to those who did not (139 (108–176) *μ*mol/L) (*P* < 0.001). Also, significant differences were observed in the various KDIGO stages at the time of AKI detection between patients with and without nephrology consultation (*P* < 0.001). Our results also show that patients with nephrology consultation had a higher proportion of patients with more severe forms of AKI (stages 2 and 3 AKI) than those without nephrology consultation at the time of detection of AKI. The severity of HA-AKI in the two groups is presented in Table 3.

The comparison on clinical outcomes between patients who had nephrology consultations/renal admission against those who did not have a nephrology consultation is summarised in Table 4. At the time of hospital discharge, higher SCr was observed in patients with nephrology consultation, (median (IQR) 152 (101–337) *μ*mol/L) than in those with no nephrology consultation (median (IQR) 101 (82–134) *μ*mol/L) (*P* < 0.001). The length of hospitalisation was longer in patients with nephrology consultation (median (IQR) 13 (6–19) days) compared to patients without consultation (median (IQR) 7 (5–12) days) (*P* = 0.003). The time from AKI occurrence to discharge was also longer in those consulted by the nephrology team than in those without (median (IQR) 10 (4–18) days versus 5 (3–9) days) (*P* = 0.001).

Overall, most patients achieved either complete or partial recovery in their kidney function during discharge; however, patients who did not have a nephrology consultation were more likely to recover (83% versus 65%, *P* = 0.022). The in-hospital mortality was not signficantly different between the two groups (11% versus 13%, *P* = 0.754). One patient with nephrology consultation required kidney replacement therapy during the AKI episode, which was continued after discharge for one month.

## 4. Discussion

This study presents evidence on the clinical characteristics of adults with HA-AKI in noncritical settings of a tertiary care university teaching hospital in Australia. Most patients in our study had mild AKI (i.e., stage 1 based on the KDIGO classification), and a small number of patients had received nephrology consultation or were admitted under the renal specialty. Nephrology consultation often occurred within the first 48 hours of AKI detection. Patients receiving a nephrology consultation were more likely to have higher SCr values at hospital presentation, at detection of AKI and at hospital discharge, experience severe forms of AKI, and had a longer length of hospitalisation than those without a nephrology consultation.

Unlike previous international studies that were conducted in ICU settings, our study provides insight into the epidemiology and characteristics of HA-AKI in conventional medical/surgical units [6, 7, 13, 21, 22]. Our study population that experienced HA-AKI was relatively older (mean ± SD age of 74.8 ± 15.8 years) and had a higher Charlson Comorbidity Index score of 3.82 (2.4) [23, 24]. The increased medical complexity and needs in older people could possibly increase the time to make treatment decisions, putting these patients at a heightened risk of developing HA-AKI. Most patients had mild AKI (92% were classified as stage 1), mainly because our study was restricted to noncritically ill patients admitted to the general wards of the hospital. Although most AKI causes were not specified or documented, our findings, consistent with those reported by other studies [22, 25], identified pre-renal AKI as the most common aetiology of AKI.

Our findings indicate that most patients with HA-AKI were not referred for nephrology consultations, and in the small number of referrals, most occurred within the first 48 hours of AKI detection. Although the KDIGO criteria provide a unique basis for epidemiologic and interventional outcome studies, these criteria are not used routinely in clinical practice. It is likely that the 48-hour window of increase in SCr for diagnosis enhances the sensitivity for earlier diagnosis relative to the 7-day window as the 48-hour window is more likely to attract the attention of clinicians. The importance of early nephrologist referral was first described by Mehta et al. where they reported that a delayed referral (≥48 hours) was associated with increased mortality and morbidity in ICU patients with kidney failure [26]. The study by Meier et al. [24], included noncritically ill patients, reported that a longer time until nephrologist referral was associated with a significantly higher mortality risk. A recent study by Park et al. [7] also reported that early consultation is associated with less likelihood of AKI being overlooked. However, their study also observed no significant change in patient mortality. Balasubramanian et al. [13] further reported that, although early nephrology consultation was associated with a reduced risk of further decrease in kidney function, it did not lead to reduced mortality. In general, implementing appropriate interventions to ensure early involvement of nephrologists in these patients may provide an opportunity for early detection of AKI incidents and thereby improve clinical outcomes [7].

Most of the baseline clinical characteristics of patients with and without nephrology consultation were comparable. However, those who received a nephrology consult tended to have a higher baseline SCr and more medical conditions than those who did not, indicating that patients potentially with underlying kidney disease and having higher medical complexity at hospital admission are important factors that could determine a clinician's perception of the need to seek specialist's advice. Baseline SCr, SCr at detection of AKI, peak SCr, and discharge SCr were overall higher in patients with nephrology consultations, implying either underlying CKD or higher AKI severity in these patients. Also, the fact that nephrology consultations were largely observed in people with stage 2 or 3 AKI (despite more than 90% of the study participants having mild AKI) indicates that SCr is a primary indicator for referral and may explain why there was a small number of referrals. We observed that nephrology consultations were sought if the AKI was more severe, potentially resulting from unrecognised HA-AKI in its early stages and patients not receiving timely interventions. Nevertheless, the results are similar to previous studies that reported that patients who received nephrology advice had higher SCr values, longer time from AKI occurrence to discharge, and longer hospitalisation than those without nephrology consultations [7, 13, 24]. This could be because patients with underlying CKD and higher medical complexity who develop HA-AKI would take longer to recover from their AKIs and are likely to have incomplete recoveries from their AKI at hospital discharge. Conversely, this could also be related to nephrology consultations predominantly occurring in patients who did not recover from their AKI episodes with standard interventions leading to increased severity of AKI, longer hospital stays, and higher SCr levels at the time of hospital discharge.

Our study did not show a significant difference in mortality based on receipt of nephrology consultation or otherwise. However, despite the predominantly mild form of AKI in our cohort, HA-AKI was associated with a considerably high in-hospital mortality (10.8%). This could relate to the relatively aged patient group observed in our study. In line with this, Selby et al. reported that advancing age was associated with an increased risk of mortality in hospitalised patients with AKI [27]. Similar studies in the past also reported comparable or higher mortality rates in hospital settings. Meier et al. [24] reported a 19.5% in-hospital mortality rate, and Bamoulid et al. [28] reported that 16% of patients with AKI died during their hospitalisation, while Wang et al. [29] reported a mortality rate of 10.8%. The mortality rate in studies that targeted patients in ICU is understandably higher—mortality rates of 20.3%, 21%, and 21.9% were reported by different studies [22, 27, 30]. However, it is essential to note that our study lacks long-term outcome data, which may have underestimated the true mortality risk in included patients. Morgera et al. and Rimes-Stigare et al. both highlighted that, compared to patients without HA-AKI, those with HA-AKI have an additional risk of mortality for at least a year beyond the kidney injury [31, 32]. The long-term risk of death was notably greater in those not achieving complete recovery of kidney function [31].

Our study has certain limitations. It is a single-centre retrospective audit, and as such, this may limit the generalisability of our results. The exclusion of patients admitted to ICU during hospitalisation meant not capturing more critical HA-AKI cases and any correlating effects on consequences, i.e., mortality and need for dialysis. However, our findings provide a basis for the need for further studies conducted locally, including on other cohorts of hospitalised patients with AKI (e.g., community-acquired AKI and patients admitted to ICU). In addition, owing to the small number of observations and retrospective nature of this study, we were unable to show, based on regression analyses, the impact of nephrology consultation on clinically important patient outcomes despite observing significant differences in clinical characteristics of AKI in patients who received nephrology consultation(s). These findings warrant further research to investigate these endpoints.

Furthermore, the retrospective nature of our study could have impacted some of our findings. For example, the definition of AKI used in this study was solely based on SCr change unaccompanied by urinary output. There was no routine electronic documentation of this, especially in patients who do not undergo routine indwelling urinary catheters. In addition, the study was only able to observe the SCr levels collected as part of the routine investigation. The current pathology system works on a requested basis for blood, and therefore, this may not provide an accurate picture of the extent, severity, and time to recovery of HA-AKI in patients that only had two SCr levels measured during hospitalisation. We studied patients until the time of discharge, and therefore, our results did not provide data on long-term outcomes. The decision to discharge patients was based on individual clinicians' judgment that can affect outcomes such as length of stay and SCr at discharge.

Finally, an adequate definition of renal recovery requires a reliable assessment of the baseline kidney function to distinguish nonrecovery from preexisting CKD. Efforts to obtain baseline kidney function within 7 days, e.g., from the primary care or referring specialists, are therefore of utmost importance. In our study, previous SCr results were not available in 118 (53.2%) patients with an elevated SCr at admission within the 12 months prior to admission and only 12 (5.4%) patients had a SCr documented within the 7 days prior to admission. This is a limitation that can impact the accuracy of determining the incidence of AKI and documentation of SCr within 7 days before admission as a baseline should be considered in routine clinical practice.

## 5. Conclusion

Our study has provided evidence on current clinical practice in the epidemiology and characteristics of HA-AKI in noncritical patients. We observed high mortality rates with HA-AKI in noncritically ill patients, which provides a strong rationale for a well-designed controlled study investigating the effect of nephrologist involvement on outcomes, such as death, length of stay, and recovery of kidney functions. Overall, our results serve as preliminary evidence for developing and implementing systematic surveillance programs, such as real-time prognostic models or an electronic AKI alerts system with automated nephrologist consultation on AKI detection.

## Figures and Tables

**Table 1:** Staging of AKI [18].

Stage	Serum creatinine
1	1.5–1.9 times baseline or ≥ 0.3 mg/dl (≥26.5 mmol/l) increase
2	2.0–2.9 times baseline
3	3.0 times baseline or increase in serum creatinine to ≥ 4.0 mg/dl (≥353.6 mmol/l)

*Note*. Adapted from Kidney Disease: Improving Global Outcomes (KDIGO) Acute Kidney Injury Work Group; KDIGO Clinical Practice Guideline for Acute Kidney Injury; *The International Society of Nephrology*; 2012; 2 (1): Table 2, Staging of AKI; p. 22.

**Table 2:** Baseline characteristics of study population at the time of AKI (*n* = 222).

Characteristics	All patients	Nephrology consultation^†^(*n* = 31)	No nephrology consultation (*n* = 191)	*P*
Age (mean (SD)) years	74.8 (15.8)	71.81 (16.8)	75.28 (15.66)	
Median (IQR)	77 (68–86)	77 (58–86)	77 (69–86)	0.339
Gender				
Male (*N* (%))	95 (42.8)	12 (38.7)	83 (43.5)	0.698
Hospitalisation				
Medical specialty (*N* (%))	194 (87.4)	28 (90.3)	166 (86.9)	0.774
Surgical specialty (*N* (%))	28 (12.6)	3 (9.7)	25 (13.1)	
Time of hospital admission to AKI (mean (SD)) days	2.5 (1.6)	2.5 (1.9)	2.5 (1.6)	
Median (IQR)	2 (1–3)	2 (1–3)	2 (1–3)	0.695
Surgical procedures during hospitalisation				
Yes (*N* (%))	36 (16.2)	4 (13)	32 (18)	0.566
Charlson's comorbidity score (mean (SD))	3.82 (2.4)	3.1 (2.7)	3.9 (2.3)	
Median (IQR)	4 (2–5)	3 (2–5)	4 (2–5)	0.322
Number of medical conditions (mean (SD))	7.2 (3.9)	7.3 (4.9)	7.1 (3.7)	
Median (IQR)	7 (4–10)	7 (4–10)	7 (4–10)	0.742
Number of medications (mean (SD))	7 (4.4)	5.1 (4.2)	7.32 (4.4)	
Median (IQR)	7 (4–10)	5 (1–8)	7 (4–11)	0.009
Risk factors for AKI (*N* (%))				
Hypertension	164 (73.9)	23 (74.2)	141 (73.8)	1
Age >75 years	126 (56.8)	18 (58.1)	108 (56.5)	1
Nephrotoxic medications^‡^	120 (54.1)	16 (51.6)	104 (54.5)	0.847
Diabetes	90 (40.5)	9 (29)	81 (42.4)	0.174
Contrast	47 (21.2)	11 (35.5)	36 (18.8)	0.055
Preexisting kidney dysfunction	35 (15.8)	8 (25.8)	27 (14.1)	0.112
Renal artery stenosis	10 (4.5)	3 (9.7)	7 (3.7)	0.149

^†^Nephrology consultation also includes patients admitted under renal medicine specialty; ^‡^angiotensin agents, i.e., angiotensin-converting enzyme inhibitors and angiotensin receptor blockers, aminoglycosides, vancomycin, and nonsteroidal anti-inflammatory drugs.

**Table 3:** Severity of AKI based on nephrology consultation.

	All patients	Nephrology consultation (*n* = 31)	No nephrology consultation (*n* = 191)	*P*
Baseline serum creatinine (SCr), *μ*mol/L (mean (SD))	103.9 (50.3)	146 (79)	97.1 (40.3)	
Median (IQR)	89.5 (69–127.3)	127 (89–175)	86 (68–121)	<0.001
SCr at point of AKI detection, *μ*mol/L (mean (SD))	155.8 (69.2)	235.1 (108.1)	142.9 (50.3)	
Median (IQR)	139.5 (109.8–181.5)	214 (157–281)	135 (105–172)	<0.001
Maximum SCr during admission, *μ*mol/L (mean (SD))	172.6 (103.5)	324.4 (179.8)	147.9 (54.6)	
Median (IQR)	147 (113.8–197)	268 (192–434)	139 (108–176)	<0.001
KDIGO stage at time of AKI (*N* (%))				
Stage 1	204 (91.9)	21 (67.7)	183 (95.8)	
Stage 2	11 (5)	5 (16.1)	6 (3.1)	
Stage 3	7 (3.2)	5 (16.1)	2 (1)	

**Table 4:** Clinical outcomes of patients with hospital-acquired AKI based on nephrology consultation.

	All patients	Nephrology consultation (*n* = 31)	No nephrology consultation (*n* = 191)	*P*
Recovery				
Complete (*N* (%))	144 (64.9)	16 (51.6)	128 (67)	0.022
Partial (*N* (%))	34 (15.3)	4 (12.9)	30 (15.7)	
No (*N* (%))	44 (19.8)	11(35.5)	33 (17.3)	
Discharge SCr, *μ*mol/L (mean (SD))	130.5 (82)	218.3 (153.1)	116.3 (51.5)	
Median (IQR)	106.5 (83.8–145.5)	152 (101–337)	101 (82–134)	<0.001
Length of hospitalisation, days (mean (SD))	10.3 (8)	14.9 (10.7)	9.5 (7.4)	
Median (IQR)	8 (5–13)	13 (6–19)	7 (5–12)	0.003
In-hospital mortality (*N* (%))	24 (10.8)	4 (12.9)	20 (10.5)	0.754
Time of AKI to hospital discharge (mean (SD)) days	7.8 (7.7)	12.4 (10.2)	7.0 (7)	
Median (IQR)	5.5 (3–11)	10 (4–18)	5 (3–9)	0.001
KDIGO stage at time of recovery/discharge (*N* (%))				
Stage 1	65 (29.3)	5 (16.1)	60 (31.4)	
Stage 2	6 (2.7)	5 (16.1)	1 (0.5)	
Stage 3	7 (3.2)	5 (16.1)	2 (0.9)	

Categorical variables are compared using Chi-square tests or Fisher-Exact test (depending on counts within individual categorisation); means of two groups are compared using the *t*-test; continuous variables are compared using the Mann–Whitney *U* test.

## Data Availability

The data used to support the findings of this study is available from the corresponding author upon request.
